# Knowledge of and attitudes towards electroconvulsive therapy (ECT) among psychiatrists and family physicians in Saudi Arabia

**DOI:** 10.1186/s12991-017-0139-1

**Published:** 2017-02-28

**Authors:** Ahmad N. AlHadi, Fahad M. AlShahrani, Ali A. Alshaqrawi, Mohanned A. Sharefi, Saud M. Almousa

**Affiliations:** 1 Department of Psychiatry, College of Medicine, King Saud University, King Saud University Medical City, PO Box 242069, Riyadh, 11322 Saudi Arabia; 2 SABIC Psychological Health Research & Applications Chair (SPHRAC), College of Medicine, King Saud University, King Saud University Medical City, PO Box 242069, Riyadh, 11322 Saudi Arabia; 3 Family Medicine Department, King Abdulaziz Medical City, National Guard, Riyadh, Saudi Arabia; 4 College of Medicine, King Saud bin Abdulaziz University for Health Sciences, Riyadh, Saudi Arabia; 5 Department of Psychiatry, King Saud University Medical City, Riyadh, Saudi Arabia; 6 Department of Emergency Medicine, Prince Sultan Military Medical City, Riyadh, Saudi Arabia; 7 Department of Internal Medicine, King Fahad Medical City, Riyadh, Saudi Arabia

**Keywords:** Electroconvulsive therapy, ECT, Psychiatrist, Family physician, Knowledge, Attitude, Saudi Arabia

## Abstract

**Objectives:**

To assess the knowledge of and attitudes towards ECT among psychiatrists and family physicians in Saudi Arabia.

**Methods:**

The study is quantitative observational cross-sectional with a convenient sample that included psychiatrists and family physicians (including residents) in Saudi Arabia.

**Results:**

Of the 434 questionnaires emailed, a total of 126 returned completed questionnaires (29% response rate). The mean age of respondents was 35 years old. Psychiatrists accounted for 68.3%. The majority were Saudis (95.2%) and male (70.6%). Around half were consultants and about two-thirds (62.7%) had worked in a facility that used ECT. Psychiatrists showed better knowledge than family physicians in their answers, with a mean total knowledge scoring of 8.12 (±1.25) out of 10 and 6.15 (±1.25), respectively (*P* < 0.0001). Among psychiatrists, 87% thought that ECT required general anesthesia, while 35% of family physicians believed so (*P* < 0.0001). Other items of ECT knowledge are discussed. Psychiatrists displayed a better attitude towards ECT than family physicians in all answers, with a mean score of 9.54 (±1.16) and 7.85 (±2.39), respectively (*P* < 0.0001).

**Conclusions:**

Psychiatrists scored better than family physicians in both knowledge and attitude regarding ECT.

## Background

Electroconvulsive therapy (ECT) is a therapeutic method used in the psychiatric field, first established in the 1930s [1]. It was proven then that ECT could be a life-saving modality, decreasing suicidal ideations and suicide attempts in severe cases of depression [2]. Some studies in Poland and Slovakia revealed that ECT is primarily indicated for affective disorders like depression [3], while schizophrenia was the main indication for ECT in eastern Europe and Asia [4]. A recent study found that ECT is used more often than medications in severe cases of depression [5]. Several studies did not find any serious side effects of ECT such as epilepsy, brain damage, and pain [6, 7]. The occurrence of permanent memory loss with ECT is uncommon [8]. Even if memory loss occurs, it usually spares emotional and personal memories [9].

Although ECT was found to be safe and effective [7], usage has declined by 80% among Hungarian psychiatrists in the past few years [10]. Also, half of Chuvash Republic psychiatrists believe that ECT is dangerous and it should be used as a last modality of treatment [4]. Additionally, only one-third of psychiatrists in Greece and Hungary would accept ECT as a treatment for themselves if they needed it [9, 10], while Romanian psychiatrists showed a more receptive attitude towards receiving ECT (47.5%) [11]. One of the reasons behind decreased use of ECT is poor knowledge [12, 13]. However, a Romanian paper found no correlation between knowledge and attitude among psychiatrists [11]. On the other hand, 86.3% of Indian psychiatrists preferred that ECT be available in general psychiatric clinics [14] and most Nigerian psychiatrists recommend using ECT when it suits their patients [12].

Non-psychiatric physicians have a more negative attitude towards ECT. Also, it was found that only 22% of non-psychiatric physicians would prefer ECT over pharmacological medications, even though they all knew that ECT would be more effective than medications. In addition, the same study showed that those non-psychiatric physicians who had not completed specialty training (i.e., general practitioners and trainees) had a more positive attitude towards ECT. Nevertheless, all still held negative attitudes [9]. Furthermore, in Nigeria, almost three-quarters of psychiatric nurses believed that ECT was helpful for most psychiatric patients [15]. A recent study found that neurologists and family practitioners needed to have more knowledge of ECT [7].

Sources of knowledge can be of a great effect on the attitude towards ECT. Media was found to be the primary source of ECT knowledge for medical and nursing students [7, 16], which usually has negative image influence on the attitude [7, 16, 17]. Poor ECT education in medical schools’ curricula may cause defect in physicians’ attitude [4]. However, clinical practice and acquiring more knowledge of ECT promote a positive attitude towards its usage [16, 18]. A study conducted with medical students in three countries (Iraq, Egypt and United Kingdom) found that the attitudes towards ECT were affected by socio-cultural factors and the modalities of education [1], while some studies revealed no socio-cultural effect on the attitude [7]. It has been observed that in many countries, there is a demand for improving education and training for ECT among students, non-psychiatric physicians, and psychiatrists themselves [9, 10].

This study aims to assess the knowledge of and attitude towards ECT among psychiatrists and family physicians in Saudi Arabia.

## Methods

The study is quantitative observational cross-sectional.

### Participants

We used a convenient sampling method to include all the psychiatrists and family physicians (including residents) in Saudi Arabia who we can reach. We were able to obtain their emails or phone numbers from the Saudi Commission for Health Specialties, hospital departments, university departments, and through colleagues. The three inclusion criteria were as follows: a psychiatrist or a family physician (including residents in training); and able to read and understand English language; and have a history of practicing medicine in Saudi Arabia.

### Questionnaire

An electronic survey was emailed and sent through WhatsApp (a messaging social media application). We used a scale that has been used in previous study, so no pilot study was done. We got the permission from the author [10].

The questionnaire consists of three sections: (1) demographic data and few questions about ECT experience; (2) knowledge of ECT (10 items with total score of 10); and (3) attitudes towards ECT (11 items with total score of 11). We decided to remove one item in the knowledge subscale which was “the efficacy of the convulsive treatment has been discovered by a Hungarian psychiatrist.” We think that this item is not necessary for Saudi psychiatrists or family physicians to know. Also, we changed “Hungary” to “Saudi Arabia” in some items to be more appropriate.

### Data analysis

Data were analyzed by the Statistical Package for Social Sciences (SPSS) [19] (Armonk, NY, USA), version 21.0. Descriptive statistical data are presented by mean values, standard deviations, and percentages. *T* tests, Chi square, and Analysis of variance (ANOVA) were used to compare subgroups. Additionally, the relationship between different variables was assessed by Pearson’s correlation. Statistical significance was set at 0.05.

## Results

### Study subjects

An online survey was sent to 435 psychiatrists and family physicians in Saudi Arabia. Completed questionnaires were returned by 126 (29% response rate). Psychiatrists comprised 68.3% (*n* = 86) of respondents; 31.7% (*n* = 40) were family physicians and general practitioners (GPs). The mean age of psychiatrists and family physicians was 34.45 (SD = 7.16) and 35.20 (SD = 7.01), respectively. The majority were male (70.6%), Saudis (95.2%), and then working in the Kingdom of Saudi Arabia (92.1%). Consultants accounted for around half of the respondents (46.8%), while 21.4% were specialists and 31.7% were residents. Most of the respondents worked in general hospitals (32.5%) followed by university hospitals (23.8%), then psychiatric hospitals (15.9%), while primary care centers accounted for only 11.9%. In addition, 15.1% worked in more than one setting. Around 44.4% of responders rated themselves as having medium knowledge of ECT, 34.1% minimal knowledge, and 21.4% a high level of knowledge. Finally, 62.7% have worked in an ECT-utilizing facility. Around 80% of psychiatrists had referred patients for ECT and would consent to receive it themselves if needed. However, among family physicians, only 5% had referred patients for ECT, and 57.5% would agree to receive it themselves (*P* ≤ 0.001). Demographic data are shown in Table 1.

**Table 1:** Demographics and personal data, ECT preferences, and experience

Item	Mean or *N* (SD or %)			*P* value
	Psychiatrists *N* = 86 (68%)	Family physicians *N* = 40 (32%)	All *N* = 126 (100%)	
Age (mean, SD)	34.45 (7.16)	35.20 (7.01)	34.69 (7.09)	0.582
Gender				
Male	62 (72.1%)	27 (30.3%)	89 (70.6%)	0.598
Female	24 (27.9%)	13 (35.1%)	37 (29.4%)	
Education				
Resident	31 (36%)	9 (22.5%)	40 (31.8%)	0.217
Specialist (registrar)	19 (22%)	8 (20%)	27 (21.4%)	
Consultant	36 (42%)	23 (57.5%)	59 (46.8)	
Saudi	80 (93%)	40 (100%)	120 (95.2%)	0.087
Non-Saudi	6 (7%)	0 (0.0%)	6 (4.8%)	
Current work country				
Inside KSA	76 (88.4%)	40 (100%)	116 (92.1%)	0.025
Outside KSA	10 (11.6%)	0 (0.0%)	10 (7.9%)	
Region				
Riyadh	60 (70%)	24 (60%)	84 (66.7%)	0.279
Outside Riyadh	26 (30%)	16 (40%)	42 (33.3%)	
Place of work				
University	23 (27%)	7 (17.5%)	30 (24%)	0.0001
General hospital	31 (36.5%)	16 (40%)	37 (29.6%)	
Psychiatric hospital	31 (36.5%)	0 (0%)	31 (24.8%)	
Primary care center	0 (0.0%)	17 (42.5%)	17 (13.6%)	
My knowledge about ECT				
Minimal	13 (15%)	30 (75%)	43 (34.1%)	0.0001
Medium	48 (56%)	8 (20%)	56 (44.5%)	
High level	25 (29%)	2 (5%)	27 (21.4%)	
Have you ever worked in an ECT-utilizing department?				
Yes	73 (85%)	6 (15%)	79 (62.7%)	0.0001
No	13 (15%)	34 (85%)	47 (37.3%)	
Have you ever referred patients to ECT?				
Yes	66 (76.7%)	2 (5%)	68 (54%)	0.0001
No	20 (23.3%)	38 (95%)	58 (46%)	
Is there any ECT-treated person in your family or among your acquaintances?				
Yes	6 (7%)	2 (5%)	8 (6.3%)	0.67
No	80 (93%)	38 (95%)	118 (93.7%)	
Do you have a psychiatric illness in your family or in your acquaintances?				
Yes	35 (40.7%)	12 (30%)	47(37.3%)	0.25
No	51 (59.3%)	28 (70%)	79 (62.7%)	
I would consent to receive ECT in case I was in a psychotic depressive condition				
Yes	72 (83.7%)	23 (57.5%)	95 (75.4%)	0.001
No	14 (16.3%)	17 (42.5%)	31 (24.6%)	

### Knowledge

Regarding questions concerning ECT knowledge, about 60% of psychiatrists answered all questions (10 questions) correctly except for one, which concerned whether long seizure duration resulted in more effective treatment. Among family physicians, 60% answered only five questions correctly out of 10 questions. Psychiatrists displayed better knowledge than family physicians in response to most questions, with a Total Knowledge Score on 10 questions of 8.12 (±1.25) and 6.15 (±1.25), respectively (*P* < 0.0001). Among psychiatrists, 87.2% thought that ECT required general anesthesia (GA) and 86% agreed that muscle relaxation is mandatory to do ECT. However, family physicians accounted for 35 and 70% on the previous two questions, respectively. Moreover, 91.9% of psychiatrists knew the number of recommended ECT weekly sessions, while only about 50% of family physicians did (*P* < 0.0001). Furthermore, about half of the family physicians agreed that ECT relieves depression faster than drugs and that it is contraindicated in patients with a history of MI, although psychiatrists scored better on both questions (74.4 and 94.2%, respectively). As for using ECT with patients aged 65 years, 88.4% of psychiatrists but only 35% of family physicians considered it acceptable (*P* < 0.0001). On the other hand, the only item family physicians answered correctly more frequently higher psychiatrists was, “The longer the seizure duration, the more effective the treatment” 87.5 versus 47.7% (*P* < 0.0001). Answers to all the items on the knowledge subscale showed a statistical significance difference between psychiatrists and family physicians except numbers 2 and 3 (Table 2).

**Table 2:** Differences between psychiatrists and family physicians in knowledge scale

Knowledge scale	Correct answer (better knowledge)			*P* value
	Psychiatrists *N* = (86)	Family physicians *N* = (40)	All *N* = (126)	
ECT is used more often in Saudi Arabia than in the USA (F)	84 (97.7%)	35 (87.5%)	119 (94.4%)	0.020
ECT has been used for the first time in the 1930s (T)	54 (62.8%)	30 (75.0%)	84 (66.7%)	0.176
The anesthetic level during ECT should be as deep as possible (F)	70 (81.4%)	29 (72.5%)	99 (78.6%)	0.257
ECT is more effective, and helps to relieve depression faster than drugs do (T)	81 (94.2%)	22 (55.0%)	103 (81.7%)	0.0001
ECT is contraindicated in patients with prior history of myocardial infarction (F)	64 (74.4%)	21 (52.5%)	85 (67.5%)	0.015
In Saudi Arabia ECT can be administered only under general anesthesia (T)	75 (87.2%)	14 (35.0%)	89 (70.6%)	0.0001
ECT can be done in Saudi Arabia without muscle relaxation (F)	74 (86.0%)	28 (70.0%)	102 (81.0%)	0.033
ECT can be used over the age of 65 (T)	76 (88.4%)	14 (35.0%)	90 (71.4%)	0.0001
The longer the seizure duration, the more effective the treatment (F)	41 (47.7%)	35 (87.5%)	76 (60.3%)	0.0001
Recommended weekly frequency of the sessions are two or three (T)	79 (91.9%)	18 (45.0%)	97 (77.0%)	0.0001
Total knowledge score: mean (SD)	8.12 (1.25)	6.15 (1.25)	7.49 (1.55)	0.0001

### Attitude

Psychiatrists showed a more positive attitude than family physicians in all questions, except for item numbers 2, 3, 4, and 7, with scores of 9.54 (±1.16) and 7.85 (±2.39) out of 11, respectively (*P* < 0.0001), as shown in Table 3. None of the responders agreed that ECT is used as a punishment. Almost all psychiatrists (96.5%) and almost three-quarters (72.5%) of family physicians believed that ECT does not cause death or permanent brain damage (*P* < 0.0001). Also, roughly 95% of psychiatrists and 80% of family physicians disagreed with the statements that ECT should be illegal and is abused by psychiatrists. However, more than half of family physicians thought that ECT is only used as a final resort and among minority populations, while roughly 30% of psychiatrists agreed with that statement. Responses to the statement “ECT is used more often for treating the poor people” showed statistical insignificance difference.

**Table 3:** Differences between psychiatrists and family physicians in Attitude scale

Attitudes scale	Correct answer (positive attitude)			*P* value
	Psychiatrists *N* = 86	Family physicians *N* = 40	All *N* = 126	
Psychiatrists often abuse ECT	81 (94.2%)	32 (80.0%)	113 (89.7%)	0.015
ECT is used to control violent patients	64 (74.4%)	29 (72.5%)	93 (73.8%)	0.820
ECT is used as a punishment	86 (100%)	40 (100%)	126 (100%)	None
ECT can cause pain	55 (64.0%)	21 (52.5%)	76 (60.35%)	0.221
ECT is dangerous and may cause death	83 (96.5%)	29 (72.5%)	112 (88.9%)	0.0001
ECT should only be used as a final resort	61 (70.9%)	16 (40.0%)	77 (61.1%)	0.001
ECT is used more often for treating the poor people	81 (94.2%)	35 (87.5%)	116 (92.1%)	0.196
ECT is used more often in minority populations	59 (68.6%)	19 (47.5%)	78 (61.9%)	0.023
ECT is an outdated, obsolete procedure	82 (95.3%)	31 (77.5%)	113 (89.7%)	0.002
ECT can cause permanent brain damage	83 (96.5%)	29 (72.5%)	112 (88.9%)	0.0001
ECT should be illegal to perform	85 (98.8%)	33 (82.5%)	118 (93.7%)	0.0001
Total attitude score	9.54 (1.16)	7.85 (2.39)	9.00 (1.82)	0.0001

### Knowledge and attitude

There was positive correlation between the mean of total knowledge score and the mean of total attitude score and it was statistically significant (*r* = 0.375, *P* < 0.0001).

## Discussion

This study assessed knowledge of and attitudes towards ECT among psychiatrists and family physicians in Saudi Arabia. Our results demonstrate that psychiatrists had better knowledge of ECT than family physicians, with the exception of one question concerning the duration of seizure. This issue is controversial. It was previously thought that “The longer the seizure duration, the more effective the treatment” is true but recent studies showed no relationship [20, 21]. Also, this difference might be explained by a better understanding of brain physiology among family physicians. Both groups in this study agreed that ECT is neither dangerous nor does it cause death. However, a recent study showed that only half of surveyed Russian psychiatrists believe that ECT is not dangerous [4]. We believe that the improvement in the education in the medical colleges in KSA has influenced the outcome. More than half of family physicians knew that ECT relieves depression faster than drugs do. Nevertheless, around three-quarters of non-psychiatric physicians in a Greek study chose medications over ECT, despite their knowledge [9]. The explanation might be that family physicians encounter more cases and they interact more often with psychiatric physicians compared to non-psychiatric physicians (surgical and non-surgical physicians) in Greece. However, psychiatrists scored better on both questions (94.2 and 74.4%, respectively). Moreover, 91.9% of psychiatrists knew the correct number of recommended ECT weekly sessions compared to around 50% of family physicians. As the psychiatrists have a chance to practice ECT, this influenced their knowledge.

Psychiatrists showed a better attitude than family physicians in all attitudes-related questions. These findings were similar to those of a study conducted in Greece [9]. This might be explained by psychiatrists utilize ECT more often and aware of its outcome than family physicians. Similar explanation was found on other studies in British Columbia and Australia [16, 18]. In this study, 83% of psychiatrists are willing to receive ECT when indicated, which shows higher attitude compared to Greek, Hungarian, and Romanian psychiatrists [9–11]. Based on these studies and the current study, having more training and experience may improve attitude. We may link this progress to poor education in the past among medical students, non-psychiatric physicians, and even psychiatrists [4, 9, 10]. One of the reasons that a small percentage of psychiatrists were unwilling to receive ECT in the past was fear of being embarrassed if their colleagues saw experiencing incontinence during the ECT session. Psychiatrists might also refuse ECT because they refused to believe they are ill [9] (Tables 4, 5).

**Table 4:** Total knowledge score

Item	Psychiatrists (mean)	*P* value	Family physicians (mean)	*P* value	All	*P* value
Age	*r* = 0.140	0.203	*r* = −0.220	0.173	*r* = −0.007	0.936
Gender						
Male	8.18	0.469	6.22	0.605	7.58	0.302
Female	7.96		6.00		7.27	
Education						
Resident	7.87	0.398	6.00	0.497	7.45	0.551
Specialist (registrar)	8.26		6.63		7.78	
Consultant	8.25		6.04		7.39	
Saudi	8.06	0.146	6.15	No comparison	7.43	
Non-Saudi	8.83		0.0^a^		8.83	0.029
Current work country						
Inside KSA	8.05	0.195	6.15	No comparison	7.40	0.018
Outside KSA	8.60		0.0^a^		8.60	
Region						
Riyadh	8.03	0.353	6.38	0.167	7.56	0.491
Outside Riyadh	8.31		5.81		7.36	
Place of work						
University	8.13	0.955	6.29	0.915	7.70	0.0001
General hospital	8.16		6.19		7.49	
Psychiatric hospital	8.07		0.0^a^		8.07	
Primary care center	0.0^a^		6.06		6.06	
My knowledge about ECT						
Minimal	7.85	0.360	6.00	0.226	6.56	0.0001
Medium	8.04		6.38		7.80	
High level	8.40		7.50		8.33	
Have you ever worked in an ECT-utilizing department?						
Yes	8.21	0.117	5.67	0.311	8.01	0.0001
No	7.62		6.24		6.62	
Is there any ECT-treated person in your family or among your acquaintances?						
Yes	8.67	0.266	5.50	0.458	7.88	0.472
No	8.08		6.18		7.47	
Do you have a psychiatric illness in your family or in your acquaintances?						
Yes	8.26	0.390	6.08	0.829	7.70	0.242
No	8.02		6.18		7.37	
I would consent to receive ECT in case I was in a psychotic depressive condition						
Yes	8.19	0.190	6.35	0.250	7.75	0.001
No	7.71		5.88		6.71	

^a^Not included in the analysis

**Table 5:** Total attitude score

Item	Psychiatrists (mean)	*P* value	Family physicians (mean)	*P* value	All	*P* value
Age	*r* = 0.190	0.081	*r* = 0.080	0.625	*r* = 0.094	0.298
Gender						
Male	9.65	0.156	7.70	0.584	9.06	0.593
Female	9.25		8.15		8.87	
Education						
Resident	9.29	0.310	7.56	0.917	8.90	0.913
Specialist (registrar)	9.58		7.88		9.07	
Consultant	9.72		7.96		9.03	
Saudi	9.56	0.421	7.85	No comparison	8.99	0.819
Non-Saudi	9.17		0.0^a^		9.17	
Current work country						
Inside KSA	9.47	0.092	7.85	No comparison	8.91	0.070
Outside KSA	10.00		0.0^a^		10.00	
Region						
Riyadh	9.60	0.430	8.13	0.380	9.18	0.120
Outside Riyadh	9.39		7.44		8.64	
Place of work						
University	9.78	0.412	8.00	0.161	9.37	0.198
General hospital	9.55		7.00		8.68	
Psychiatric hospital	9.36		0.0^a^		9.36	
Primary care center	0.0^a^		8.59		8.59	
My knowledge about ECT						
Minimal	8.92	0.116	7.57	0.216	7.98	0.0001
Medium	9.65		8.25		9.45	
High level	9.64		10.50		9.70	
Have you ever worked in an ECT-utilizing department?						
Yes	9.70	0.001	9.00	0.205	9.65	0.0001
No	8.62		7.65		7.92	
Is there any ECT-treated person in your family or among your acquaintances?						
Yes	10.00	0.123	6.50	0.420	9.13	0.842
No	9.50		7.92		8.99	
Do you have a psychiatric illness in your family or in your acquaintances?						
Yes	9.77	0.116	7.17	0.242	9.11	0.615
No	9.37		8.14		8.94	
I would consent to receive ECT in case I was in a psychotic depressive condition						
Yes	9.60	0.259	7.78	0.839	9.16	0.146
No	9.21		7.94		8.52	

^a^Not included in the analysis

Sources of knowledge can greatly influence the attitude towards ECT. Media and poor medical school curricula have a negative impact on the attitude towards and knowledge of ECT [7, 16, 17]. The media, especially in movies, represent ECT in a wrong way, depicting it as torture that destroys memories. A short psychiatry rotation and inadequate clinical exposure contribute to poor knowledge and attitude [1]. This may lead to poor knowledge among future psychiatrists as this previously affected the psychiatrists in Texas and Nigeria [12, 13]. Even though a study showed no link between knowledge and attitude [11], we believe that knowledge of ECT plays a major role. In this study, we noticed that the psychiatrists group had a higher knowledge score compared than the other group, which may reflect on their high attitude score also. This is supported by the significant positive correlation between knowledge and attitude in this study. Furthermore, another possible factor is the socio-cultural environment, which has a great effect on this society. This was observed in another study in three other countries [1]. Yet another paper was done in Turkey on medical students, psychology students, and lay people found no significance [7]. In our study, the questionnaire that we adapted did not have the option of “I do not know,” which may have affected the participants’ response accuracy.

## Conclusion

We concluded that psychiatrists have better knowledge and attitude towards ECT than Family Physicians. From our observation, there is a correlated relation between the knowledge and attitude.

## Limitations

We recommend including the option “I do not know” in the answers to improve the accuracy of participants’ responses.

## Abbreviations

ECT: electroconvulsive therapy
GA: general anesthesia
GPs: general practitioners
ANOVA: analysis of variance
KSA: Kingdom of Saudi Arabia
